# Inhibition of RNF182 mediated by Bap promotes non-small cell lung cancer progression

**DOI:** 10.3389/fonc.2022.1009508

**Published:** 2023-01-06

**Authors:** Yating Liu, Lianlian Ouyang, Chao Mao, Yuanbing Chen, Na Liu, Ling Chen, Ying Shi, Desheng Xiao, Shuang Liu, Yongguang Tao

**Affiliations:** ^1^ Department of Pathology, Xiangya Hospital, Central South University, Key Laboratory of Carcinogenesis and Cancer Invasion, Ministry of Education, Hunan, Changsha, China; ^2^ National Health Commission (NHC) Key Laboratory of Carcinogenesis (Central South University), Cancer Research Institute and School of Basic Medicine, Central South University, Changsha, Hunan, China; ^3^ Postdoctoral Research Station of Clinical Medicine & Department of Hematology and Critical Care Medicine, The 3rd Xiangya Hospital, Central South University, Changsha, China; ^4^ Department of Oncology, Institute of Medical Sciences, National Clinical Research, Center for Geriatric Disorders, Xiangya Hospital, Central South University, Changsha, Hunan, China; ^5^ Hunan Key Laboratory of Early Diagnosis and Precision Therapy in Lung Cancer, Second Xiangya Hospital, Central South University, Changsha, China

**Keywords:** RNF182, Bap, AhR, NSCLC, methylation

## Abstract

**Introduction:**

Ubiquitylation that mediated by ubiquitin ligases plays multiple roles not only in proteasome-mediated protein degradation but also in various cellular process including DNA repair, signal transduction and endocytosis. RING finger (RNF) proteins form the majority of these ubiquitin ligases. Recent studies have demonstrated the important roles of RNF finger proteins in tumorigenesis and tumor progression. Benzo[a]pyrene (BaP) is one of the most common environmental carcinogens causing lung cancer. The molecular mechanism of Bap carcinogenesis remains elusive. Considering the critical roles of RNF proteins in tumorigenesis and tumor progression, we speculate on whether Bap regulates RNF proteins resulting in carcinogenesis.

**Methods:**

We used GEO analysis to identify the potential RING finger protein family member that contributes to Bap-induced NSCLC. We next used RT-qPCR, Western blot and ChIP assay to investigate the potential mechanism of Bap inhibits RNF182. BGS analyses were used to analyze the methylation level of RNF182.

**Results:**

Here we reported that the carcinogen Bap suppresses the expression of ring finger protein 182 (RNF182) in non-small cell lung cancer (NSCLC) cells, which is mediated by abnormal hypermethylation in an AhR independent way and transcriptional regulation in an AhR dependent way. Furthermore, RNF182 exhibits low expression and hypermethylation in tumor tissues. RNF182 also significantly suppresses cell proliferation and induces cell cycle arrest in NSCLC cell lines.

**Conclusion:**

These results demonstrated that Bap inhibits RNF182 expression to promote lung cancer tumorigenesis through activating AhR and promoting abnormal methylation.

## Introduction

Lung cancer is the leading cause of cancer-related deaths worldwide. Non-small cell lung cancer (NSCLC), including adenocarcinoma (LUAD) and squamous cell carcinoma (LUSC), is the most common type of lung cancer (1, 2). A large amount of somatic mutations, abnormally inactivated tumor suppressor genes and activated oncogenic factors contribute to lung carcinogenesis (3, 4).

Benzo(a)pyrene (Bap) is a member of the polycyclic aromatic hydrocarbon (PAH) family and Bap is usually produced when organic matters incompletely burned. Cigarette smoke and well-done barbecued-meat contain high levels of Bap and are common sources of general population Bap exposure (5, 6). Bap and its key metabolite benzo(a)pyrene-7,8-diol-9,10-epoxide (BPDE), form adducts with DNA and lead to mutagenic and carcinogenetic effects. Although Bap is recognized as one of the most common environmental pollutants and carcinogens causing lung cancer (6), the molecular mechanism of Bap carcinogenesis remains elusive.

Bap is a potent natural ligand of AhR which is a ligand-activated transcription factor of the basic helix-loop-helix/Per-Arnt-Sim family. Once binding to its ligands, AhR translocates into the nucleus and regulates a battery of gene expression. Activation of AhR is one of the mechanisms responsible for adverse effects of Bap (7–11).

Ubiquitin-protein enzymes (E3s) are of particular concern in ubiquitination, as they not only transfer activated ubiquitin from ubiquitin-conjugating enzymes to protein substrates but also confer substrate specificity (12). Recently, the RING finger protein family, a complex set of E3s that contain RNF domain, was demonstrated to play important roles in tumorigenesis and tumor progression (13–16). Considering the critical roles of RNF proteins, we speculate on whether Bap regulates RNF proteins resulting in carcinogenesis. Hence, we screened the RNF family genes which expression were altered in Bap key metabolite BPDE-treated lung fibroblasts cells through GEO database (GSE19510) and found that RNF182 was decreased significantly in a dose-dependent way. Next, the biological function experiments of RNF182, including cell proliferation, colony formation and cell cycle phase distribution demonstrated that inhibition of RNF182 promotes NSCLC progression. Moreover, the low expression of RNF182 is significantly associated with patients poor clinical outcome. These results suggested that RNF182 may function as a tumor suppressor in NSCLC and inhibition of RNF182 may contribute to NSCLC inition and progression.

On the other hand, we revealed that RNF182 was inhibited by Bap through both AhR-mediated regulation and DNA methylation. These findings may clarify the mechanism of Bap-mediated abnormal downregulation of RNF182 in NSCLC and identify new diagnostic, prognostic and therapeutic schedule in NSCLC.

## Materials and methods

### Ethics approval and consent to participate

This study was conducted at the Cancer Research Institute and the Third Xiangya Hospital, Central South University, Hunan, China. All of the protocols were reviewed and approved (No.2021-S055) by the IRB of Third Xiangya Hospital, Central South University and performed in accordance with national guidelines.

### Data acquisition and databases

National Center for Biotechnology Information Gene Expression Omnibus (GEO) (https://www.ncbi.nlm.nih.gov/geo/) is a public functional genomics database that are provided to help users query and download experiments and curated gene expression profiles. Search was conducted using “Benzo(a)pyrene and lung” key words. Series GSE19510 (Dataset: GDS3706) provided the expression profiling of lung WI-38 fibroblasts exposed to BPDE (17).

The Cancer Genome Atlas (TCGA) (https://www.cancer.gov/) is a public funded project that aims to catalogue and discover major cancer-causing genomic alterations to create a comprehensive “atlas” of cancer genomic profiles (18).

Gene Expression Profiling Interactive Analysis (GEPIA) (http://gepia.cancer-pku.cn/) is a newly developed interactive web server for analyzing the RNA sequencing expression data tumors and normal samples from the TCGA and the GTEx projects, using a standard processing pipeline (19). Entering gene name “RNF182”, choosing “Expression on Box Plots” parts, and selecting LUAD and LUSC datasets to get the expression of RNF182 in LUAD and LUSC datasets.

The UCSC (https://genome.ucsc.edu/) genome browser database is a free website that that allows scientists to visualize gene expression and metadata annotation distribution throughout a single-cell dataset or multiple datasets. The CpG islands of RNF182 were obtained by searching RNF182 in the genome browser part in UCSC database.

### Cell lines and plasmids

The human lung bronchus cell line HBE (CRL- 2741) and lung carcinomatous cell lines A549 (CCL-185) were obtained from American Type Culture Collection (University Boulevard, Manassas, VA, USA). The human lung carcinomatous cell line PC9 was stored in the Department of Molecular Biology, Cancer Research Institute (Central South University, Hunan, China). The cell lines were characterized by mycoplasma detection, DNA fingerprinting, isozyme detection, and cell viability by the provider. HBE and PC9 were cultured in Roswell Park Memorial Institute 1640 Media (Gibco) supplemented with 10% FBS, penicillin, and streptomycin. A549 cells were cultured in Dulbecco’s Modified Eagle’s Medium/Nutrient Mixture F-12 (Gibco) supplemented with 10% FBS, penicillin, and streptomycin.

Cells were seeded about 30% density in 6 well plate. After cells growing adherence for 24 hours, cells were exposed to Bap at different times (24h, 48h and 72h) and concentration (10µM, 20µM and 40µM) and finally collected for Western blot and RT-qPCR analyses. For BGS analysis, cells were seeded about 30% density in 6 well plate and continuously exposed to vehicle control (dimethyl sulfoxide, DMSO), Bap (Sigma) or ITE (Targetmol, USA) after 24 hours cell culture. Bap were freshly added to cells each time after overnight cell attachment (40µM,72h). ITE were freshly added every 8 hours and cell media were changed (10µM, 8h*3).

AhR shRNA vectors and control (GV248) were purchased from GeneChem (www.genechem.com.cn). Lentiviral shRNA clones targeting human RNF182 (#4 TTGCTAGGTTTGCTCTACTTC; #5 CCTCGTTATTCTTATGGTGTA) and the nontargeting control construct were purchased from GeneChem. Lentiviral particles were produced in 293T cells. A549 and PC9 cell lines that were transfected and selected with puromycin at a concentration of 2 μg/ml.

### Western blot and antibody

The cells were washed with PBS and harvested by scraping and centrifugation at 500 g for 5 min. The harvested cells were washed with PBS and lysed for 30 min in the IP lysis buffer with proteinase inhibitor. Then the soluble fraction was collected by centrifugation at 12,000 g for 15min. Protein lysates were quantified and resolved on SDS-PAGE gels, transferred to PVDF membranes (Millipore) and immunoblotted with primary antibody, followed by incubation with a secondary antibody. Antibody signals were detected using an enhanced chemiluminescence system. The following antibodies were used: RNF182 (Abcam, #ab156127, 1:500), β-actin (Proteintech, #66009-1-lg, 1:1000), AhR (Santa Cruz, sc-5579).

### Cell proliferation and colony formation

Details of these procedures have been previously described (20, 21). Briefly, for cell proliferation, 1000 cells were seeded into 96-well plates and incubated with CellTiter 96 AQueous One Solution Cell Proliferation Assay (Promega #G3581) (MTS: media=1:9) for 1 hour in indicated times. After incubation, the absorbance at 490 nm was measured by multimode reader (Bio-TeK, EL800).

For the colony formation assay, 500 cells were seeded into 6-well plates in appropriate media. After 2 weeks, the cells were washed with PBS, fixed in methanol and stained with crystal violet. Colonies were counted using ImageJ (1.47 v, NIH, USA).

### Cell cycle analysis

Details of cell cycle analysis have been previously described (20). Briefly, PC9 cells were harvested and washed with PBS and fixed in 70% ice-cold ethanol overnight. After centrifugation at 800 rpm for 5 min, cells were washed with ice-cold PBS and resuspended in 0.5 mL PBS containing PI (50 μg/mL) and RNase (100 μg/mL) for 15 min in the dark. Subsequently, the cell cycle distribution was analyzed by flow cytometry (BD Biosciences, FACS Canto II).

### Real-time quantitative PCR

RNA was extracted from cell lines using RNAiso Plus (Takara) according to the manufacturer’s protocol. cDNA was synthesized by RNA (1 μg) using a Prime-Script™ RT Reagent Kit with gDNA Eraser (Takara). Amplification and semiquantification of transcripts were performed using FastStart Universal SYBR Green Master Mix (Roche) and specific primers on a 7500 Fast Real Time PCR System (Applied Biosystems, Life Technologies). Amplification was performed at 95°C for 10 min, followed by 40 cycles of 15 s at 95°C and 1 min at 60°C. Fold change in gene expression was calculated using the 2−ΔΔCt method. mRNA expression was normalized against β-actin, allowing comparison of mRNA levels. The primers used were list as below:

RNF182 (F: GTCCTCCAACTGCCTGGTCATA; R: GTGGTGACAGAGTCGAAACTCG); RNF141 (F: TCCTCTGTAACATCTTGTCAGGC; R: CTGTGAGCACAAGGCAGGATGA); RNF130 (F: GCCAGTTACCATGACTCATCCAG; R: GGCTGAAGTTCTTCGGTGGCAT); RNF110 (F: CACTATCGTGGAGTGCCTGCAT; R: GGTTTTATGGACCTGCACGTCAC); RNF150 (F: TCAGCCGTGGTCATCTTCAACG; R: CGGTGATGTTTCTTTCCAGCAGG); RNF24 (F: GTGTGCCTAGAAGACTTCAAGCC; R: GCTGTAGAACTGGCATGTTGCAC); RNF220 (F: ACCGATGACCTCCACCATTCAG; R: CTGCCCTTCATCTTGCTTCCTC); RNF11 (F: GCCTAAAGGAGTTTATGACCCTG; R: GTCCAGGTGATAGATGTGCATGC); RNF121 (F: GTGCAGACTACATGGCATCTACC; R: GATTGCAGGACAGCCTATACGTG); RNF7 (F: AGGCGACAAGATGTTCTCCCTC; R: TCAGCTTGACATCTAAGACAGGC); RNF181 (F: GACTGCCATTGAGATGCCTTGC; R: GCCTTATCTCGTCTGTGCTCCT); RNF26 (F: CAGGACCATCAGAGTGACACCT; R: GCAACACTGTCTTGCTCTGGTC); RNF34 (F: GGAACTGGTAGAGAAAGTAAACCG; R: ACACAGGCTGTCGTCTTCCTCA); CYP1A1 (F: GATTGAGCACTGTCAGGAGAAGC; R: ATGAGGCTCCAGGAGATAGCA0G);

### Chromatin immunoprecipitation assays

ChIP assays were performed essentially as described previously (20, 21). Breifly, 10 cm dish cells (90% confluence) were fixed with formaldehyde (1% final volume concentration, Sigma) for 10 min at room temperature. Fixation was stopped by addition of 1/10 volume 1.25 M glycine and incubated for 5 min at room temperature. Sonication was performed using a Qsonica sonicator (6 min, 20 s on, 20 s off), and 400 μg protein-chromatin complex was used for each chromatin immunoprecipitation. Antibody against AhR (Santa Cruz, sc-5579), H3K27ac (Proteintech, 39135) or normal rabbit IgG (Cell signaling technology #2729S) as a negative control were used for ChIP assays. Antibody-protein complexes were captured using Protein A/G Mix Magnetic Beads (Millpore). ChIP DNA was analyzed by qPCR using FastStart Universal SYBR Green Master Mix (Roche) in an ABI-7500 instrument (Applied Biosystems) using primers:

(RNF182-ChIP-F: CAGCGCCGTAGAGACAAAGC; RNF182-ChIP-R: GGCTGCGGCGGCGCCTGGGAG).

### Bisulfite genomic sequencing of DNA

Genomic DNA was extracted from A549 cells, PC9 cells, NSCLC carcinoma tissues and adjacent tissues using ONE-4-ALL Genemic DNA Mini-Preps Kit (Bio Basic Canada INC, B618503) and treated with sodium bisulfite using a Zymo DNA Modification kit (Zymo Research, D5030T). The amplified fragments were subcloned into the T-vector (Takara, 6013). The primers #1 (F: TTTAGGGTTTATGGTAGATGT TTAG, R: AAATATAATCTCACTCCACCCAAC) and primers#2 (F: GGTTGGGTGG AGTGAGATTATATT, R: CCACACCTTTCTCCTATAAAACTATACC) were used for subcloned. Six colonies were chosen randomly and were sequenced using the Primer RV-M by Sangon Biotech.

### Statistical analyses

Results are shown as the mean ± SEM or SD. Significant differences between two groups were analyzed by unpaired Student’s t-test (two-tailed) if data were normally distributed; otherwise, data were analyzed by Kolmogorov–Smirnov test. All statistical analyses were performed using Prism 8.0 GraphPad software. A p-value < 0.05 was considered statistically significant.

## Results

### Bap inhibits the mRNA and protein level of RNF182

**Figure 1:**
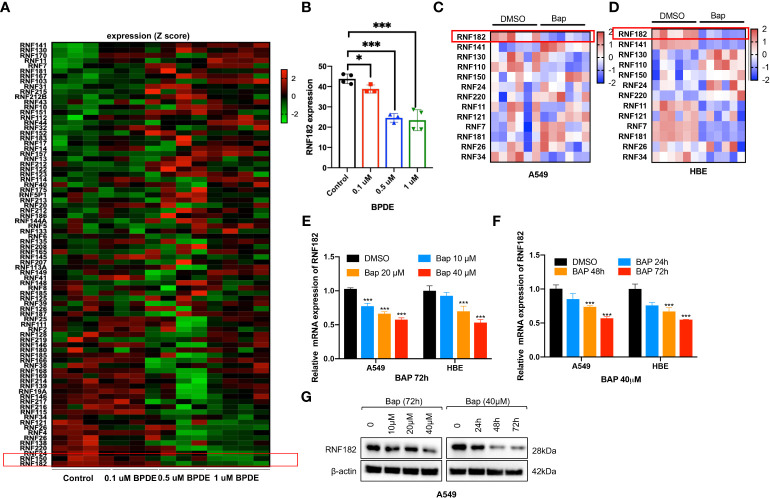
Bap inhibits the mRNA and protein level of RNF182 in LUAD cells. **(A)** GEO database (GSE19510) showed that BPDE inhibits mRNA expression of RNF182 in a dose-dependent manner in normal human lung WI-38 fibroblasts. **(B)** Quantitative results of RNF182 in A. (mean± SD, n=4). p-values are indicated (*p < 0.05, ***p < 0.001) in each panel (two-sided Student’s t-test). BPDE exposure groups were compared to the control group. **(C, D)** Heatmap of RNF family members mRNA expression in A549 and HBE cells treated with Bap (40µM, 72h). **(E)** RT-qPCR shows the mRNA levels of RNF182 in A549 and HBE cells in a dose-dependent manner in response to BaP. (mean± SD, n=3). p-values are indicated (***p < 0.001) in each panel (two-sided Student’s t-test). Bap exposure group were compared to the control group. **(F)** RT-qPCR shows the mRNA levels of RNF182 in A549 and HBE cells in a time-dependent manner in response to BaP. (mean± SD, n=3). p-values are indicated (***p < 0.001) in each panel (two-sided Student’s t-test). Bap exposure group were compared to the control group. **(G)** Western blot shows the levels of RNF182 protein in A549 cells in a time- and dose-dependent manner in response to BaP.

To identify the potential RING finger protein family member that contributes to Bap-induced NSCLC, we analyzed the relative expression of RING finger protein family member in GEO database (GSE19510) of gene expression profiles of lung fibroblasts cells WI-38 (17), and found that RNF182 was significantly downregulated by BPDE in a dose-dependent way (**Figures 1A, B**). Next, Bap was prepared and used to treat HBE (normal lung epithelial cells) and A549 (lung adenocarcinoma) cells, and the results showed that treatment of the cells with Bap significantly downregulated mRNA expression of RNF182 in both HBE and A549 cells (**Figures 1C, D**). We further confirmed that Bap downregulated RNF182 at mRNA levels in dose- and time-dependent manners in HBE and A549 cells (**Figures 1E, F**). Consistently, Western blot analysis confirmed that Bap downregulated RNF182 protein levels in dose- and time-dependent manners in A549 cells (**Figure 1G**).

### RNF182 expression is decreased in tumor tissues in NSCLC

**Figure 2:**
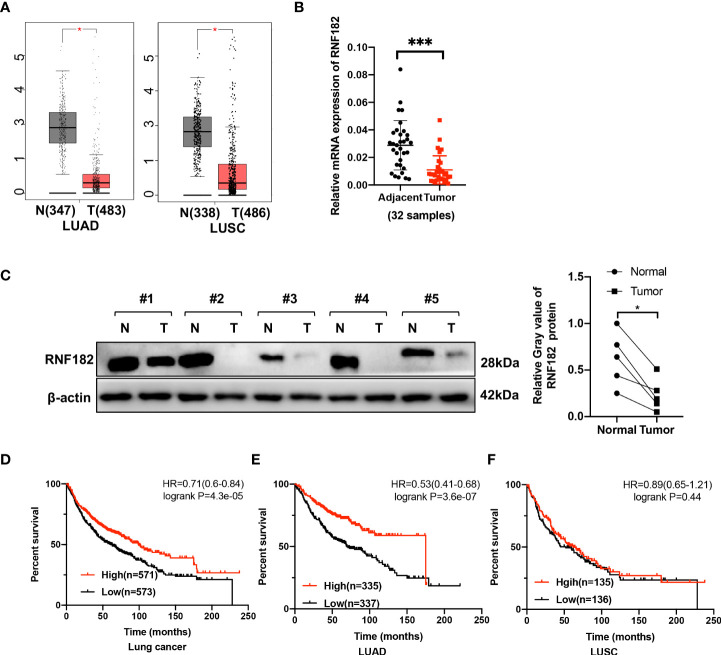
RNF182 expression is decreased in tumors tissues and associated with prognosis in LUAD patients. **(A)** Box plot showed RNF182 expression (transcript per million, TPM) is decreased in LUAD and LUSC tumor tissue. Data were adapted from Gepia. **(B)** Relative mRNA expression of RNF182 in LUAD patients (mean± SD, n=32). p-values are indicated (***p < 0.001) in each panel (two-sided Student’s t-test). **(C)** RNF182 protein level in LUAD tumor tissues and adjacent tissues (n=5). p-values are indicated (*p < 0.05) in each panel (two-sided Student’s t-test). **(D, E)** Kaplan-Meier survival curves showing that patients with low expression of RNF182 had a worse prognosis than patients with high expression of RNF182 in lung cancer and LUAD patients. **(F)** Kaplan-Meier showing that RNF182 expression and prognosis in LUSC patients.

To explore the expression of RNF182 in NSCLC, we examined the relative level of RNF182 in LUAD tissues (n=483) compared with normal tissues (n=347) and LUSC tissues (n=486) compared with normal tissues (n=338) through Gepia database (http://gepia.cancer-pku.cn). We found that RNF182 expression was significantly decreased in both LUAD and LUSC samples compared with the normal tissues (**Figure 2A**). Moreover, we detected the mRNA level (n=32) and protein level (n=5) of RNF182 in NSCLC tissues compared with adjacent normal tissues (**Figures 2B, C**). RNF182 expression was significantly decreased in NSCLC tumor tissues. Next, through K-M plotter analysis, we observed that low expression of RNF182 was associated with poor survival in LUAD patients (logrank P=4.3e-05) and lung cancer patients (logrank P=3.6e-07) (**Figures 2D, E**). However, there were no differences in survival between the two groups in LUSC patients (**Figure 2F**).

Taken together, these observations revealed that RNF182 expression is inhibited in NSCLC tumor tissues and that its low expression in NSCLC patients is associated with poor clinical outcome, indicating that NSCLC progression is associated with RNF182 alteration.

### RNF182 inhibits malignant progression of NSCLC

**Figure 3:**
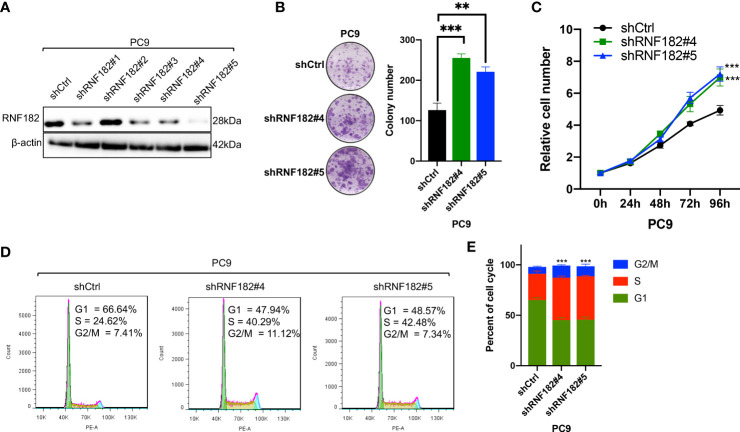
RNF182 inhibits malignant progression of NSCLC. **(A)** Western blot analysis was conducted to detect RNF182 protein levels in PC9 cells stably transfected with distinct RNF182 shRNA expression vectors or a control vector (shCtrl). **(B)** Colony formation assay of PC9 cell lines. (mean± SD, n=32). p-values are indicated (**p < 0.01, ***p < 0.001) in each panel (two-sided Student’s t-test). **(C)** MTS assay of PC9 cell lines. (mean ± SD, n=5). p-values were obtained by two-way ANOVA (***p < 0.001). **(D)** PI-FACS analysis showing that RNF182 affects cell cycle progression in PC9 cells. **(E)** Quantification of PI-FACS analysis. (mean± SD, n=3). p-values are indicated (***p < 0.001) in each panel (two-sided Student’s t-test).

To further uncover the physiological role of RNF182 in NSCLC, we depleted expression of RNF182 using independent shRNA hairpins in PC9 cells that RNF182 is high expressed (**Figure 3A**). RNF182 knockdown significantly increased cell colony formation (**Figure 3B**) and cell proliferation (**Figure 3C**). Furthermore, we also found that RNF182 knockdown reversed the induction of cell cycle arrest in G1 phase (**Figures 3D, E**).

These results suggested that RNF182 significantly inhibits NSCLC tumorigenesis and growth *in vitro*.

### Aryl hydrocarbon receptor-mediates Bap-inhibited RNF182

**Figure 4:**
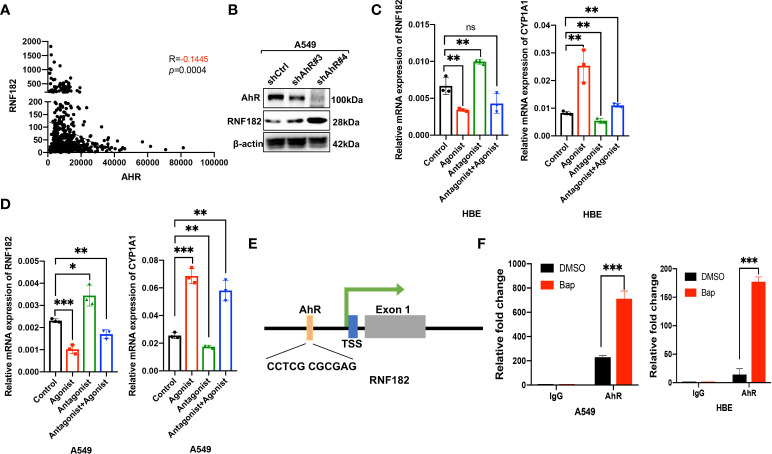
Aryl hydrocarbon receptor-mediates Bap- inhibited RNF182. **(A)** The correlation between AhR and RNF182 mRNA expression in LUAD patients (n=594). Data are adapted from TCGA. R=-0.1445, p=0.0004. **(B)** Western blot analysis showing the expression of RNF182 in AhR knockdown cell lines. **(C, D)** Relative mRNA expression of RNF182 and CYP1A1 in HBE **(C)** and A549 **(D)** cells treated with AhR agonist and Antagonist. (mean± SD, n=3). p-values are indicated (*p < 0.05, **p < 0.01, ***p < 0.001, ns means “no significance”) in each panel (two-sided Student’s t-test). Each group was compared to the control group. **(E)** The AhR binding site is located at the upstream of the RNF182 transcription start site (TSS) **(F)** ChIP assay was performed using BaP-treated or untreated HBE and A549 cells. (mean± SD, n=3). p-values are indicated (***p < 0.001) in each panel (two-sided Student’s t-test).

Aryl hydrocarbon receptor (AhR) mediates Bap-induced gene expression like CXCL13 and SMARCA6 in lung epithelia cells (22, 23). Bap triggered the activation of AhR *via* nuclear relocation of AhR (23). Interestingly, a significant negative correlation was observed between RNF182 and AhR in NSCLC patient samples (n=594, *p*=0.0004, R=-0.1445) based on TCGA database (**Figure 4A**). To further examine the regulation of AhR in RNF182, we knocked down AhR with two separate short hairpin RNAs (shRNAs) in A549 cell line that AhR is high expressed. The results demonstrated that knockdown of AhR in A549 cells increased RNF182 expression (**Figure 4B**). AhR agonist also inhibited RNF182 at mRNA level and AhR antagonist reversed the inhibition of AhR agonist in RNF182. CYP1A1, a well-known target gene of AhR, was used to confirm agonist and antagonist effects (**Figures 4C, D**). Chromatin immunoprecipitation (ChIP) were conducted to test AhR and RNF182 interaction, and the results showed that Bap treatment promoted the enrichment of AhR in the RNF182 promoter, indicating that Bap decreases RNF182 expression *via* promoting the recruitment of AhR to RNF182 promoter (**Figures 4E, F**).

Taken together, our results demonstrated that RNF182 was downregulated by AhR, which functions as a transcription factor and inhibits RNF182 expression by binding to its promoter region.

### RNF182 is hypermethylated in NSCLC tumor tissues

**Figure 5:**
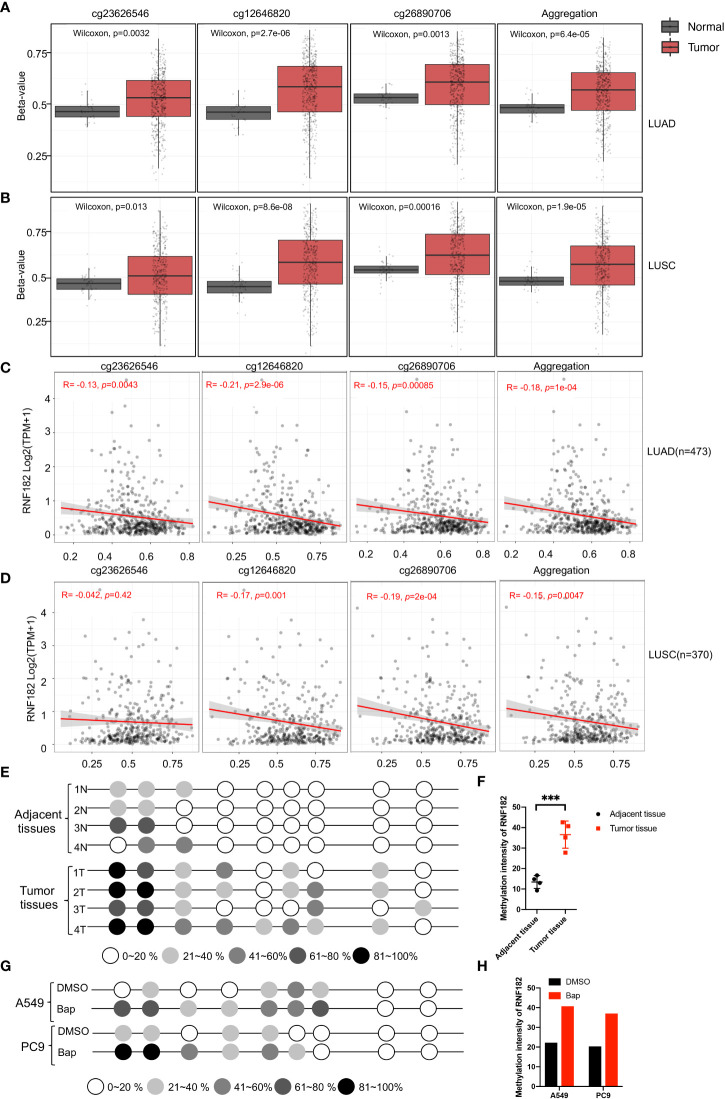
Methylation of RNF182 in LUAD and LUSC samples. **(A, B)** Several probes detected methylation level of RNF182 in LUAD **(A)** and LUSC **(B)**. The data are adapted from SMART App, a web application for comprehensively analyzing the DNA methylation data of TCGA project. **(C, D)** RNF182 expression and methylation shows a negative correlation in LUAD **(C)** and LUSC **(D)**. The data are adapted from SMART App, a web application for comprehensively analyzing the DNA methylation data of TCGA project. **(E)** Methylation status of RNF182 CpG sites in NSCLC tumor tissues (n=4) and adjacent tissues assayed by bisulfate genomic sequencing (BGS). The degree of methylation was calculated by the number of methylated colonies in each CpG site divided by the total number of colonies in the same CpG sites (n=6). **(F)** Methylation intensity and of RNF182 in NSCLC tumor and adjacent tissues (mean± SD, n=4). p-values are indicated (***p < 0.001) in each panel (two-sided Student’s t-test). **(G)** Methylation status of RNF182 CpG sites in A549 cells and PC9 cells treated with Bap (40µM, 72h) /DMSO assayed by bisulfate genomic sequencing (BGS).The degree of methylation was calculated by the number of methylated colonies in each CpG site divided by the total number of colonies in the same CpG sites (n=6). **(H)** Methylation intensity and of RNF182 in A549 cells and PC9 cells.

A CpG island was observed at the promoter region of RNF182 in the UCSC database. Abnormal DNA methylation has been found in cancer cells, especially at silenced tumor suppressor genes (24). Next, we tested whether downregulation of RNF182 in tumor tissues was caused by DNA methylation. Aberrant hypermethylation in a CpG island of RNF182 was detected in LUAD and LUSC tissues compared with normal tissues *via* several probes through Shiny Methylation Analysis Resource Tool (SMART) (**Figures 5A, B**). Moreover, an evident anticorrelation between RNF182 expression levels and methylation intensity was observed in these samples (**Figures 5C, D**). We further analyzed RNF182 CpG sites methylation in more details by using bisulfite genomic sequencing (BGS) analysis. Consistently, extensive methylation was detected in tumor tissues (n=4), whereas low levels of methylation were observed in adjacent tissues (n=4) (**Figures 5E, F**).

As Bap exposure has been reported to alter gene methylation in previous studies (25–28), we next detected the methylation level of RNF182 in A549 and PC9 cells treated with Bap by using bisulfite genomic sequencing analysis to confirm whether Bap exposure could alter RNF182 methylation. Interestingly, when treated with Bap, aberrant hypermethylation of RNF182 was observed *via* BGS analysis (**Figures 5G, H**).

**Figure 6:**
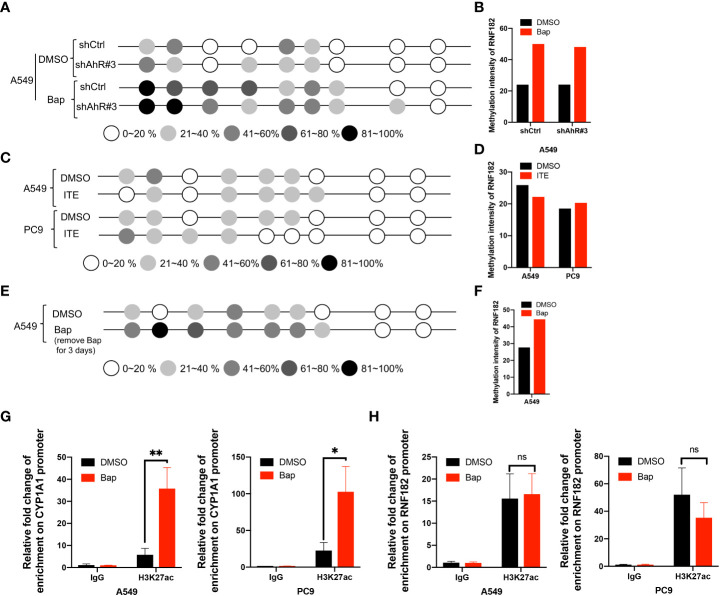
Bap mediates-RNF182 hypermethylation through an AhR-independent way. **(A)** Methylation status of RNF182 CpG sites in A549 shCtrl cells and shAhR#3 cells treated with Bap (40µM, 72h) /DMSO assayed by BGS.The degree of methylation was calculated by the number of methylated colonies in each CpG site divided by the total number of colonies in the same CpG sites (n=6). **(B)** Methylation intensity and of RNF182 in A549 shCtrl cells and shAhR#3 cells. **(C)** Methylation status of RNF182 CpG sites in A549 cells and PC9 cells treated with ITE (10µM, 8h*3) /DMSO assayed by BGS. The degree of methylation was calculated by the number of methylated colonies in each CpG site divided by the total number of colonies in the same CpG sites (n=6). **(D)** Methylation intensity and of RNF182 in A549 and PC9 cells treated with ITE (10µM, 8h*3) /DMSO. **(E)** Methylation status of RNF182 CpG sites in A549 cells assayed by BGS. A549 cells were treated with Bap (40µM) for 72h and were cultured for 72h after remove Bap from the media. The degree of methylation was calculated by the number of methylated colonies in each CpG site divided by the total number of colonies in the same CpG sites (n=6). **(F)** Methylation intensity and of RNF182 in A549 cells that were treated with Bap (40µM) for 72h and were cultured for 72h after remove Bap from the media. **(G)** ChIP assay was performed showing the enrichment of H3K27ac on the promoter of CYP1A1in DMSO/Bap-treated A549 and PC9 cells. (mean± SD, n=3). p-values are indicated (*p < 0.05, **p < 0.01) in each panel (two-sided Student’s t-test). **(H)** ChIP assay was performed showing the enrichment of H3K27ac on the promoter of RNF182 in DMSO/Bap-treated A549 and PC9 cells. (mean± SD, n=3). p-values are indicated (ns means “no significance”) in each panel (two-sided Student’s t-test).

To further confirm whether AhR contributes to Bap-mediates aberrant hypermethylation of RNF182, we detected the methylation level of RNF182 in A549 knockdown AhR cells treated with Bap by BGS analysis. We found that A549 AhR knockdown cells also exerts hypermethylation when treated with Bap (**Figures 6A, B**), which indicates that Bap mediates RNF182 hypermethylation in an AhR-independent way. We also used anther AhR agonist-ITE, a putative endogenous AHR ligand, to confirm the aberrant hypermethylation of RNF182 caused by AhR activation or Bap metabolic activation. As shown in **Figures 6C, D**, the methylation intensity of RNF182 showed similar levels both in A549 and PC9 cells, suggesting that AhR activation does not contribute to hypermethylation of RNF182. To test the effect of Bap on the gene methylation in a transient or in a persistent way, we treated A549 cells with Bap (40µM) for 72h and were cultured for 72h after remove Bap from the media. The BGS analysis showed that even Bap was removed from the culture media, the hypermethylation of RNF182 was still observed in Bap treated cells (**Figures 6E, F**), indicating that Bap effects on gene methylation may be a persistent way.

As Bap has been reported to alter histone H3 posttranslational modifications (29), a key mechanism of epigenetics, we next investigated whether Bap addition changes the histone acetylation patterns in the CYP1A1 gene and the RNF182 gene. It was found that CYP1A1 and RNF182 promoter region were dramatically enriched with H3K27ac through UCSC database. To confirm this, we conducted ChIP assay with the use of anti‐H3K27ac antibody. ChIP assay showed that Bap promotes the enrichment of H3K27ac on the promoter of CYP1A1 both in A549 and PC9 cells (**Figure 6G**), but does not promote the enrichment of H3K27ac on the promoter of RNF182 (**Figure 6H**).

Collectively, the above results demonstrated that RNF182 is downregulated in NSCLC through its hypermethylation mediated by Bap through an AhR independent way.

## Discussion

In this study, we provided evidence that RNF182 plays a critical role in suppressing NSCLC progression. Our findings suggested that RNF182 exerts a tumor suppressor effect in NSCLC through inhibiting cell growth and promoting cell cycle arrest that are key characteristics of cancer progression (30). Moreover, we found that RNF182 expression is significantly downregulated in LUAD tumor tissues compared with that in matched adjacent tissues, and the downregulation of RNF182 is strongly correlated with poor prognosis in LUAD patients. The reduced expression is linked closely to promoter methylation, as confirmed by methylation analyses in clinical tissues through public database and bisulfate genomic sequencing, indicating that promoter methylation is the principal regulatory mechanism of RNF182 inactivation in NSCLC.

RNF182, a member of RING E3 ubiquitin ligase family, has previously been reported to associate with innate immune system (31), cancers (20), Alzheimer’s disease (32), ischemia-reperfusion injury (33) and various biological functions such as nuclear factor-kappa B (NF-κB) (31)and apoptosis (34). For example, multiple studies have demonstrated that RNF182 could bind with p65 and increase its ubiquitination and degradation (20, 31, 35), which is a well-known transcription factor of NF-κB, to modulate cell proliferation and inflammatory response (31). RNF182 also contributes to myocardial ischemia-reperfusion injury (MIRI) *via* inhibiting mTOR signaling pathway (33). Considering the important physiological functions and tumor suppressive effects of RNF182, further study of the upstream regulatory mechanism of RNF182 will undoubtedly be helpful to the treatment of NSCLC.

Cigarette smoke is the leading cause of the lung cancer-related death, accounting for more than 87% (14, 36). Cigarette smoke and environmental pollutants contains high level of Bap, which is a member of the polycyclic aromatic hydrocarbon (PAH) family (6, 37–39). Accumulating evidence indicates that Bap exposure contributes to inducing cancer stem cell (CSC)-like property (40), immune destruction (36) and cell malignant transformation (6, 39). However, the underlying mechanisms of how Bap exposure induces epigenetics dysregulation in NSCLC has been poorly understood.

AhR is a ligand-activated transcriptional factor that regulates divers process, including malignant transformation, hematopoietic cell development, and fate determination of immune cell lineages (36, 41–43). Recent studies showed that Bap mediates target genes expression through inducing the translocation of AhR from cytoplasm to nucleus (9, 23, 44). Here, we reported that RNF182 is a target of AhR, and the expression level of RNF182 is inhibited by Bap through the AhR signaling pathway. Bap promotes AhR binding to the promoter of RNF182 and finally results to the decreased expression of RNF182. These results demonstrated that Bap-mediated AhR signaling is critical for promoting cancer progression and tumorigenicity in NSCLC cells.

Recent studies have shown that BaP can adversely modify human epigenetic characteristics, leading to health disorders (45–48). Several studies used Bap and its key metabolite BPDE to study modulation of DNA methylation *in vitro*. BPDE was shown to bind to DNA, which resulted in the methylated DNA formation and alteration of DNA methyltransferase (DNMT) (46, 47). Consistently, other two studies have described BaP-induced hypo- and hypermethylation *in vitro* cell line models (48, 49). Our studies demonstrated that Bap induced RNF182 aberrant hypermethylation in A549 and PC9 cell lines, and the decreased expression of RNF182 is associated with aberrant hypermethylation. This study suggested that Bap inhibits the expression of RNF182 to promote NSCLC progression.

The mechanisms responsible for DNA methylation response to Bap exposure are complex and not well understood. One of the most studied mechanisms of Bap-induced aberrant promoter methylation is BPDE-DNA adducts formation (48, 50, 51). Other mechanism such as Bap-mediated disruption of DNA methyltransferases (52, 53) has been also proposed to be involved in changing DNA methylation. Oxidative stress induced by Bap exposure could also influence the expression of DNA methyltransferase. Our study demonstrated that Bap exposure induces hypermethylation of RNF182 promoter in an AhR independent way as knockdown of AhR did not rescue the hypermethylation of RNF182 caused by Bap exposure. And ITE, an endogenous AhR ligand, also exerts no effects on the methylation patterns of RNF182. These results suggested that AhR may not be involved in aberrant DNA methylation of RNF182 caused by Bap exposure. The mechanism of Bap effects on the hypermethylation of RNF182 remains to be addressed in the future studies.

In addition to inducing hypermethylation of RNF182, Bap could activate AhR to suppress the expression of RNF182. AhR is involved in the control of many genes upon recognition of its binding motifs to regulate diverse process, including malignant transformation, hematopoietic cell development, and fate determination of immune cell lineages (43, 54). AhR could function as a transcriptional promoter or a transcriptional suppressor. For example, AhR suppressed ILC2s expression and functions through the counteraction of Gfil (a positive regulator of ILC2s) at the promoter but enhanced ILC3 maintenance to protect the host from citrobacter rodentium infection (55). Moreover, a study focused on cardiac differentiation demonstrated that TCDD impairs human embryonic stem cell cardiac differentiation by promoting AhR binding and repression of key mesoderm genes (56). Our results also showed that Bap can activate AhR binding to inhibit RNF182 expression. Hence, Bap induced suppression of RNF182 through hypermethylation in an AhR independent way, and transcriptional regulation in an AhR dependent way.

However, the specific mechanisms underlying the anti-tumor role of RNF182 have not been thoroughly elucidated to date, and further investigations are needed. What’s more, the mechanism of Bap effects on the hypermethylation of RNF182 remains to be addressed in the future studies. And Bap activates AhR to repress RNF182 expression, the potential transcriptional factor that inhibited by AhR also need further studies.

In conclusion, this study demonstrated that RNF182 functions as a tumor suppressor *via* suppressing NSCLC cells proliferation and inducing cell cycle arrest. Furthermore, RNF182 is inactivation and hypermethylation in NSCLC tumor tissues. Further molecular mechanism research revealed that AhR is activated by Bap in NSCLC cell lines and its activation decreases the expression of RNF182. In addition, Bap exposure induces hypermethylation of RNF182, leading to the suppression of RNF182. These findings imply that RNF182 plays an important role in suppressing NSCLC and Bap exposure significantly increases NSCLC risk through inhibiting the expression of RNF182.

## Data availability statement

The raw data supporting the conclusions of this article will be made available by the authors, without undue reservation.

## Ethics statement

The studies involving human participants were reviewed and approved by the IRB of Third Xiangya Hospital (No.2021-S055), Central South University. The patients/participants provided their written informed consent to participate in this study.

## Author contributions

YT, YS, DX, and SL designed/planned the study and wrote the paper. YL performed experiments using the three types of cells and analyzed data. LO, CM, LC and NL participated in writing the paper. YC and YT participated in discussion of related experiments. YL and LO performed experiments and analyzed data. All authors contributed to the article and approved the submitted version.
